# Peptides as Quorum Sensing Molecules: Measurement Techniques and Obtained Levels *In vitro* and *In vivo*

**DOI:** 10.3389/fnins.2017.00183

**Published:** 2017-04-12

**Authors:** Frederick Verbeke, Severine De Craemer, Nathan Debunne, Yorick Janssens, Evelien Wynendaele, Christophe Van de Wiele, Bart De Spiegeleer

**Affiliations:** ^1^Drug Quality and Registration Group, Faculty of Pharmaceutical Sciences, Ghent UniversityGhent, Belgium; ^2^Department of Nuclear Medicine, AZ GroeningeKortrijk, Belgium; ^3^Department of Nuclear Medicine and Radiology, Faculty of Medicine and Health Sciences, Ghent UniversityGhent, Belgium

**Keywords:** quorum sensing peptides, analytical methods, reporter bacteria, biosensors, chromatography, microbiome

## Abstract

The expression of certain bacterial genes is regulated in a cell-density dependent way, a phenomenon called quorum sensing. Both Gram-negative and Gram-positive bacteria use this type of communication, though the signal molecules (auto-inducers) used by them differ between both groups: Gram-negative bacteria use predominantly *N*-acyl homoserine lacton (AHL) molecules (autoinducer-1, AI-1) while Gram-positive bacteria use mainly peptides (autoinducer peptides, AIP or quorum sensing peptides). These quorum sensing molecules are not only involved in the inter-microbial communication, but can also possibly cross-talk directly or indirectly with their host. This review summarizes the currently applied analytical approaches for quorum sensing identification and quantification with additionally summarizing the experimentally found *in vivo* concentrations of these molecules in humans.

## Introduction

The idea of bacteria being single isolated organisms has been outdated for many years. In the late 1960s, a cell-density dependent bioluminescence was observed in the marine symbiotic bacterium *Vibrio fisheri* (Nealson et al., 1970; Nealson and Hastings, 1979). This cell-density dependent regulation of gene expression is defined as quorum sensing and consists of at least four steps: (I) synthesis of signal molecules, called autoinducers, (II) excretion of the signal molecules, (III) at a certain threshold concentration, activation of a specific receptor and as a result (IV) activation or suppression of gene expression (Sifri, 2008). For example, with the increase of the number of *Vibrio fisheri* bacteria, the amount of autoinducer in the external environment reaches a certain level and triggers the production of the enzyme luciferase resulting in bioluminescence (Engebrecht and Silverman, 1984). The genes involved in quorum sensing, are responsible for activities that are only of use when performed by a large number of cells, for example: bioluminescence, antibiotic production, formation of biofilms, and production of virulence factors (Rutherford and Bassler, 2012).

Both Gram-negative and Gram-positive bacteria apply quorum sensing for communication, but they produce different auto-inducers. Gram-negative bacteria mainly depend on *N*-acyl homoserine lacton (AHL) molecules (autoinducer-1, AI-1) while Gram-positive bacteria use modified oligopeptides (autoinducer peptides, AIP) (Taga and Bassler, 2003). These peptides possess a large structural diversity and frequently undergo post-translational modifications (Sturme et al., 2002). A third type of autoinducers are boron-furan-derived signal molecules (autoinducer-2, AI-2) and are produced and detected by both Gram-negative and Gram-positive bacteria (Li and Nair, 2012). Besides these 3 main groups, there is also a fourth group of miscellaneous quorum sensing molecules (Barber et al., 1997; Flavier et al., 1997; Holden et al., 1999; Pesci et al., 1999; Higgins et al., 2007; Wei et al., 2011).

In this review, the focus will be on the production of quorum sensing molecules belonging to the first three groups, i.e., AHL molecules, AI-2 molecules and quorum sensing peptides.

**Figure 1:**
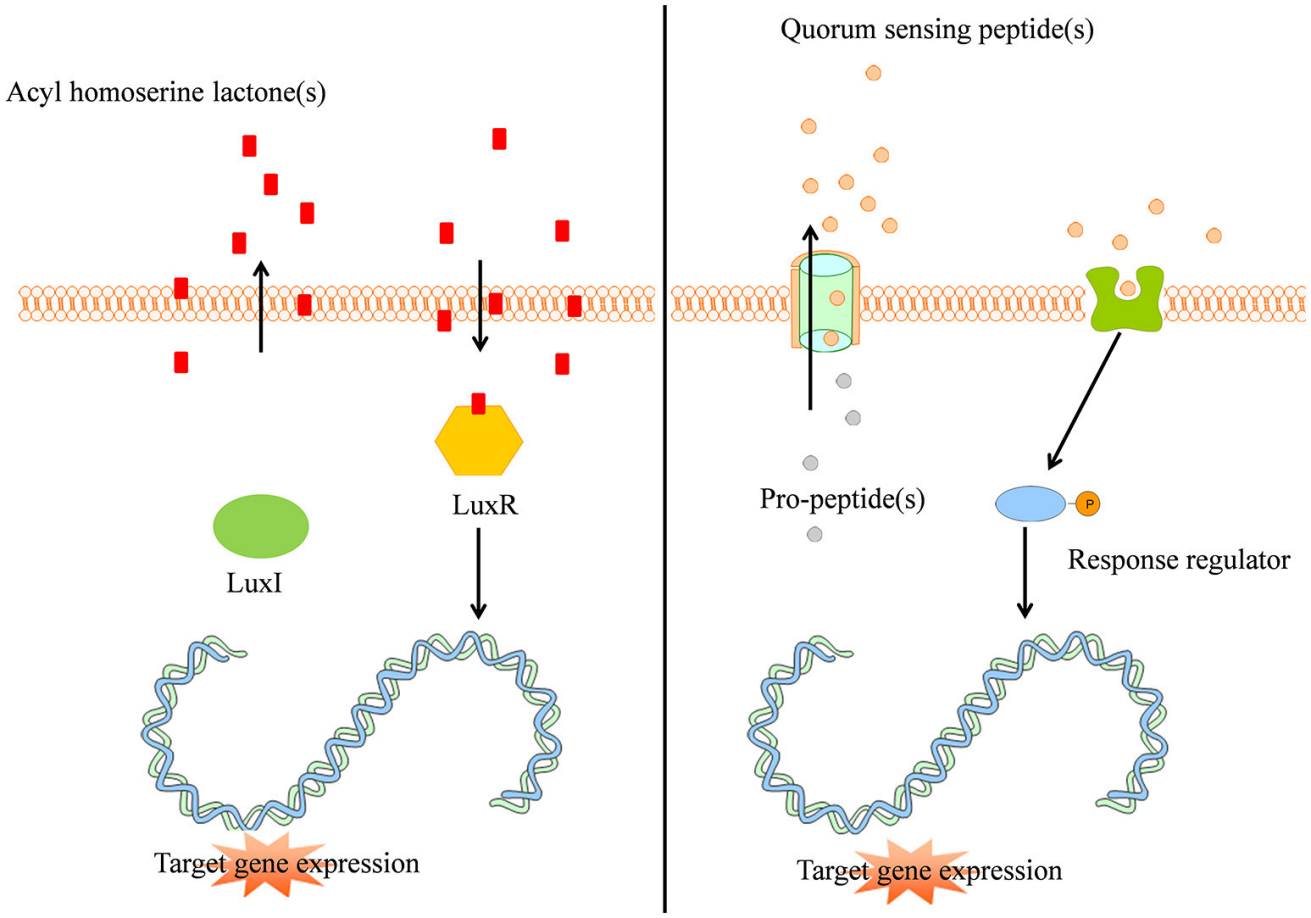
**(Left panel)** Typical Gram-negative quorum sensing mechanism. Acyl homoserine lactone molecules, synthesized by LuxI, passively pass the bacterial cell membrane and when a sufficient concentration is reached (threshold level) activate the intracellular LuxR which subsequently activates target gene expression in a coordinated way. **(Right panel)** Quorum sensing peptides are synthesized by the bacterial ribosomes as pro-peptidic proteins and undergo posttranslational modifications during excretion by active transport. The quorum sensing peptides bind membrane associated receptors which get autophosphorylated and activate intracellular response regulators via phosphor-transfer. These phosphorylated response regulators induce increased target gene expression.

The quorum sensing mechanism of Gram-negative bacteria can be described using the example of *Vibrio fisheri*: an intracellular autoinducer synthase (LuxI) synthesizes AHL signal molecules by catalyzing a reaction between S-adenosylmethionine and an acyl carrier protein (Sifri, 2008; Rutherford and Bassler, 2012). Due to the small size and lipophilicity of AHL autoinducers, they readily pass the cell membrane by means of passive diffusion (Sifri, 2008). If the concentration of AHL is sufficiently high, the AHL autoinducer binds to the intracellular LuxR protein and provokes the LuxR DNA binding domain to reveal. Subsequently, the LuxR protein binds to DNA, causing activation of target gene transcription (Figure 1 left panel, Rutherford and Bassler, 2012). More than 100 Gram-negative bacterial species apply a LuxI/LuxR-type system with an autoinducer synthase (e.g., LuxI) and a transcriptional regulator (e.g., LuxR, Sifri, 2008).

**Figure 2:**
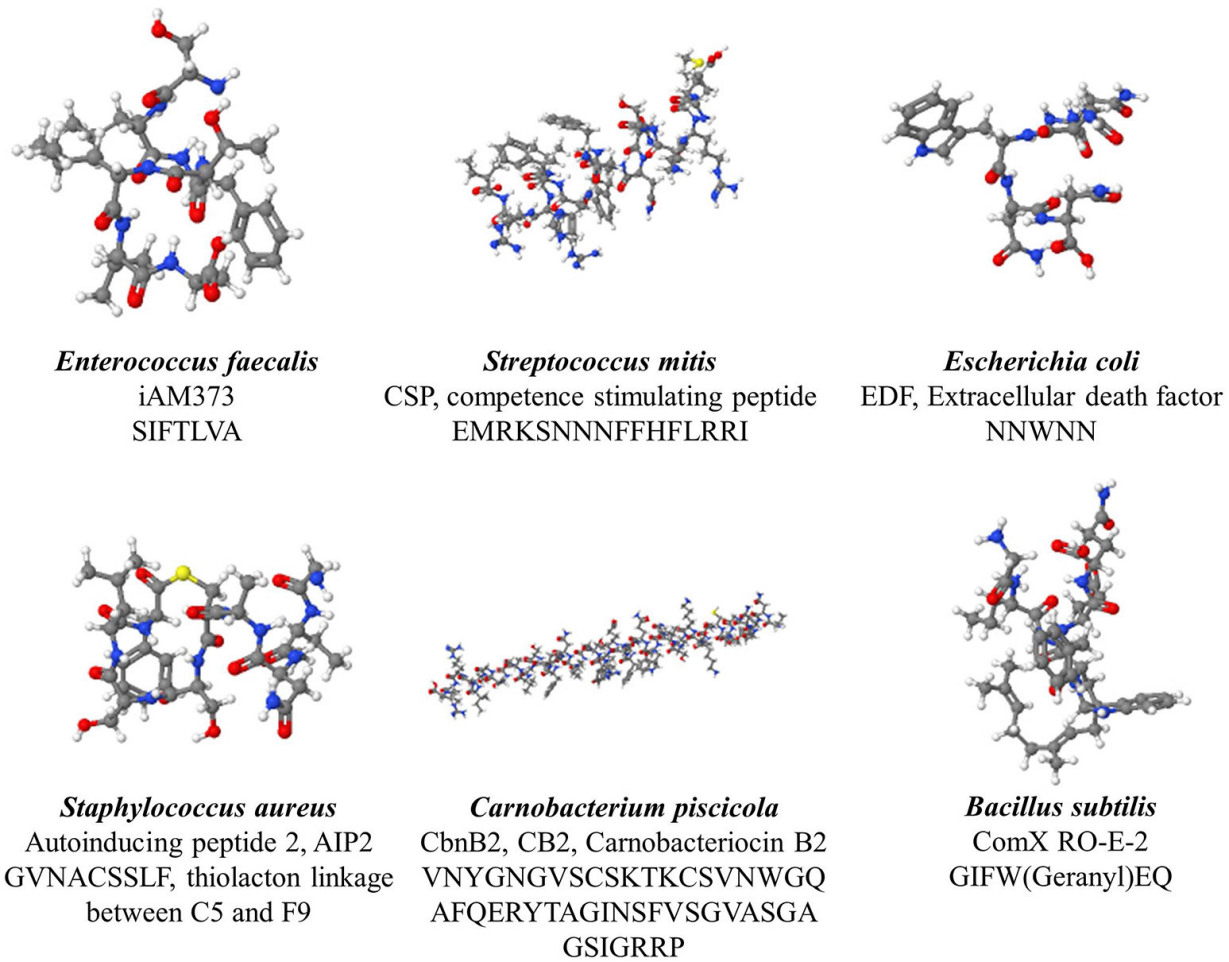
**Various quorum sensing peptides, illustrating the structural diversity observed among quorum sensing peptide**.

Gram-positive bacteria apply peptides as autoinducers for quorum sensing (Figure 1 right panel). Examples of this heterogeneous group of peptides are given in Figure 2. These peptides are synthesized by ribosomes as precursor peptides and undergo posttranslational modifications during excretion to become activated and stabilized (Sturme et al., 2002). In general, the secretion of the AIP is facilitated by a membrane associated ATP-binding cassette (ABC) transporter (Sturme et al., 2002). As the population density increases, the AIPs accumulate in the environment. When a certain threshold level is reached, binding of an AIP to a receptor initiates activation of the receptor kinase by phosphorylation on a conserved histidine residue (Bassler, 1999; Sturme et al., 2002). Subsequently, the activated receptor kinase transfers the phosphoryl group to a conserved aspartate residue of the intracellular response regulator, which in turn will be activated (Bassler, 1999). The activated response regulator influences the transcription of target genes, including the AIP genes, genes for the receptor kinase and response regulator and genes for the ABC transporter. Based on the species, the nomenclature of the quorum sensing mechanisms can be different, due to the involved genes and receptor(s). For example, *Staphylococci* species employ the *agr*-quorum sensing system, *Streptococci* species employ the *ComX*-quorum sensing system and *Bacilli* species use the *Rap*-quorum sensing system.

The current mainstream opinion is that the AIPs only serve as inter-microbial communication molecules, but other biological actions are known and being explored as well, for example the lantibiotic nisin peptide of *Lactococcus lactis*, also has known antimicrobial properties (Sturme et al., 2002). The influence of quorum sensing peptides on human tumor progression has recently also been investigated (De Spiegeleer et al., 2015; Wynendaele et al., 2015a).

Quorum sensing molecules do not only serve as intra-species communication molecules. It has been demonstrated that certain AHLs are secreted and recognized by several species of Gram-negative bacteria, hereby potentially acting as “cross-talk” signals (interspecies communication). Another indication is the presence of AI-2 in both Gram-positive and Gram-negative bacteria. Co-culture systems of *V. harveyi* and *E coli* have indicated that AI-2 production by one species affects gene expression in the other (McNab and Lamont, 2003; Ryan and Dow, 2008; Pereira et al., 2013). For example, cell culture supernatant of *P. aeruginosa* is capable of mediating cell death of *E. coli* via a quorum sensing mediated process (Kumar et al., 2013).

## Inter-kingdom communication

Inter-kingdom communication, due to co-evolution, between bacteria and eukaryotes is readily known (Lyte and Ernst, 1993). The knowledge of the inter-kingdom communication between bacteria and the human host has extended remarkably during recent years [reviewed in references Hughes and Sperandio, 2008; Karavolos et al., 2013; Clarke et al., 2014; O'Mahony et al., 2015; Kendall and Sperandio, 2016; Moos et al., 2016; Oleskin et al., 2016; Stilling et al., 2016]. For example, to modulate the expression of virulence factors, bacteria have shown the remarkable capacity to monitor neuroendocrine hormones produced by the host, e.g., adrenaline and noradrenaline (Lyte, 1993, 2004; Lyte and Ernst, 1993; Sperandio et al., 2003; Freestone et al., 2008; Karavolos et al., 2008, 2011; Pacheco and Sperandio, 2009; Spencer et al., 2010; Lyte et al., 2011). For example, Enterohaemorrhagic *E. coli* (EHEC) is able to modify its mobility and virulence expression in relationship to adrenaline and noradrenaline concentration (Rasko et al., 2008; Pacheco and Sperandio, 2009). Moreover, the mouse gut microbiome itself is capable of producing catecholamines, thus possessing the ability to interact with the host (Asano et al., 2012). The neurotransmitter γ-aminobutyric acid (GABA) produced by various *Lactobacilli* plays an important role in our brains (Barrett et al., 2012; Clarke et al., 2014). Another important group of messenger molecules in the gut-brain communication are the bacterial short chain fatty acids, fermentation products of bacterial metabolism as these molecules are capable of triggering peptide YY release (Holzer et al., 2012). Bacteria are also capable of sensing a variety of human peptide hormones, e.g., somatostatin (Yamashita et al., 1998) and gastrin (Chowers et al., 1999). Both bacteria and humans apply cyclic dipeptides for communication purposes and hence these molecules might be of extreme interest for the gut-brain communication axis [opinionated in reference (Belleza et al., 2014)]. It has also been demonstrated that some quorum sensing peptides are capable to penetrate the blood-brain barrier in a mouse model, without significant subsequent efflux from the brain (Wynendaele et al., 2015b). The Gram-negative quorum sensing molecules, i.e., *N*-acylhomoserinelactones (AHLs), easily pass eukaryotic cell membranes, due to their lipophilic nature (Fuqua et al., 1994).

Immunomodulation by odHL (*N*-3-oxo-dodecanoyl-L-homoserine lactone) has been reported (Pritchard, 2006a; Teplitski et al., 2011) and occurs in a TLR4-independent manner (Kravchenko et al., 2006). The activity of macrophages (Gomi et al., 2006; Kravchenko et al., 2006, 2008; Miyairi et al., 2006; Thomas et al., 2006), epithelial cells (Kravchenko et al., 2006, 2008; Jahoor et al., 2008; Cooley et al., 2010), mast cells (Li et al., 2009), fibroblasts (Kravchenko et al., 2006, 2008; Miyairi et al., 2006; Jahoor et al., 2008), T-lymphocytes (Wagner et al., 2007), B-lymphocytes (Telford et al., 1998a), and neutrophils (Zimmermann et al., 2006) is influenced by odHL. The outcome of the immunomodulatory properties of odHL are not straight-forward, as some studies suggest a proinflammatory response (Smith et al., 2001, 2002a,b; Vikström et al., 2005; Thomas et al., 2006; Jahoor et al., 2008; Mayer et al., 2011), whereas others point out an anti-inflammatory effect (Telford et al., 1998a; Chhabra et al., 2003; Hooi et al., 2004; Kravchenko et al., 2008; Skindersoe et al., 2009), thus facilitating persistent infection. As a rationale to elucidate this apparent discrepancy, a concentration-dependent effect has been proposed (Teplitski et al., 2011; Pritchard, 2006a). Besides odHL, *P. aeruginosa* also produces an aromatic quorum sensing molecule, i.e., 2-amino-acetophenon. This molecule contributes to the establishment of a chronic infection by damping the inflammatory immune response (Bandyopadhaya et al., 2012). A third quorum sensing molecule from *P. aeruginosa*, 2-heptyl-3-hydroxy-4(1H)-quinolone, has shown to inhibit T cell proliferation (Pritchard, 2006b), possibly via the IL-2 receptor pathway (Hooi et al., 2004). As a direct effect of its immunomodulating properties, odHL evokes IL-8 production on fibroblasts and bronchial epithelial cells, resulting in an accelerated apoptosis by mammalian macrophages and neutrophils (DiMango et al., 1995; Smith et al., 2002a; Tateda et al., 2003). Growth of human colorectal and prostate cancer cells is inhibited by odHL and analogs (Dolnick et al., 2005; Oliver et al., 2009), whereas down-modulation of STAT3 by odHL induces apoptosis in breast carcinoma cells (Li et al., 2004). Additionally, quorum sensing molecules produced by the Gram-negative *P. aeruginosa* in nosocomial urinary tract infection (UTI) might also be accountable for renal damage. As an opportunistic pathogen, *P. aeruginosa* especially affects immunocompromised patients and treatment is further hindered by its ability to form biofilms on urinary catheters (Gupta et al., 2013). Immunomodulation by these quorum sensing molecules can also have implications via the neuroendocrine-immune system axis, which is frequently involved in various diseases (Procaccini et al., 2014).

The clinical significance of the microbiome-host relationship is becoming increasingly apparent. Differences between the microbiome of healthy individuals and cancer patients have been related to susceptibility to cancer (Bultman, 2014), e.g., an increase in fecal *E. coli* has been associated with development of colon cancer (Wynendaele et al., 2015a). Strong indications have been found that changes in the microbiome are not only associated with tumorigenesis, but also directly contribute to tumorigenesis (Zackular et al., 2013; Baxter et al., 2014). The microbiome has been shown to alter the tumor-environment by influencing the host's neuroendocrine system (Erdman and Poutahidis, 2014). *Helicobacter pylori* is, besides causing gastro-duodenal ulcers, accountable for the majority of stomach cancers (Ernst and Gold, 2000). Moreover, the association between inflammatory bowel diseases, e.g., chronic ulcerative colitis and Chrohn's disease, and colon carcinoma is widely accepted (Balkwill and Mantovani, 2001). The microbiome is relevantly involved in alterations of the human immune system and host's metabolism (Thaiss et al., 2016). Quorum sensing molecules also affect human health via quorum sensing mediated biofilms (Kalia, 2013) which are associated with a wide array of infections (Beloin et al., 2014). These infections contribute to an increase in morbidity, mortality, and public costs (Haas et al., 2014). Alterations in the human microbiome have also been linked to several central nervous disorders, e.g., autism (Finegold et al., 2010; Tomova et al., 2015), depression (Naseribafrouei et al., 2014; Jiang et al., 2015), and schizophrenia (Castro-Nallar et al., 2015). The gut microbiome is likely associated with various conditions via the gut-brain axis, e.g., anxiety and altered neurochemistry (Cryan and O'Mahony, 2011) and increasing evidence points toward the influence of the microbiome in neuroendocrine diseases, e.g., as a mediator in diabetes mellitus via the cholinergic nervous system (Parekh et al., 2016). Additionally, aging has also been linked to alterations of the gut microbiome, which are, among others, related to immunosenescence and immune aging (De Spiegeleer et al., 2016; Mello et al., 2016).

## Analytical techniques

A variety of analytical techniques have been reported in literature for the qualitative and quantitative analysis of quorum sensing molecules. Generally, these techniques can be considered as chromatographic/mass spectroscopic techniques or based on biosensor systems using (genetically modified) reporter bacteria and will be discussed in more detail in the following sections regarding the detection and quantification of quorum sensing molecules in human biological samples and bacterial cell culture broths.

### Biosensor systems using reporter bacteria

In these systems, the presence of a compound of interest is qualitatively and/or quantitatively detected based on a signal produced by the bacterial reporter. The produced signal can be colorimetric, fluorimetric, bioluminescent, chemiluminescent, turbidimetric, colony forming units to name a few (van der Meer and Belkin, 2010).

**Figure 3:**
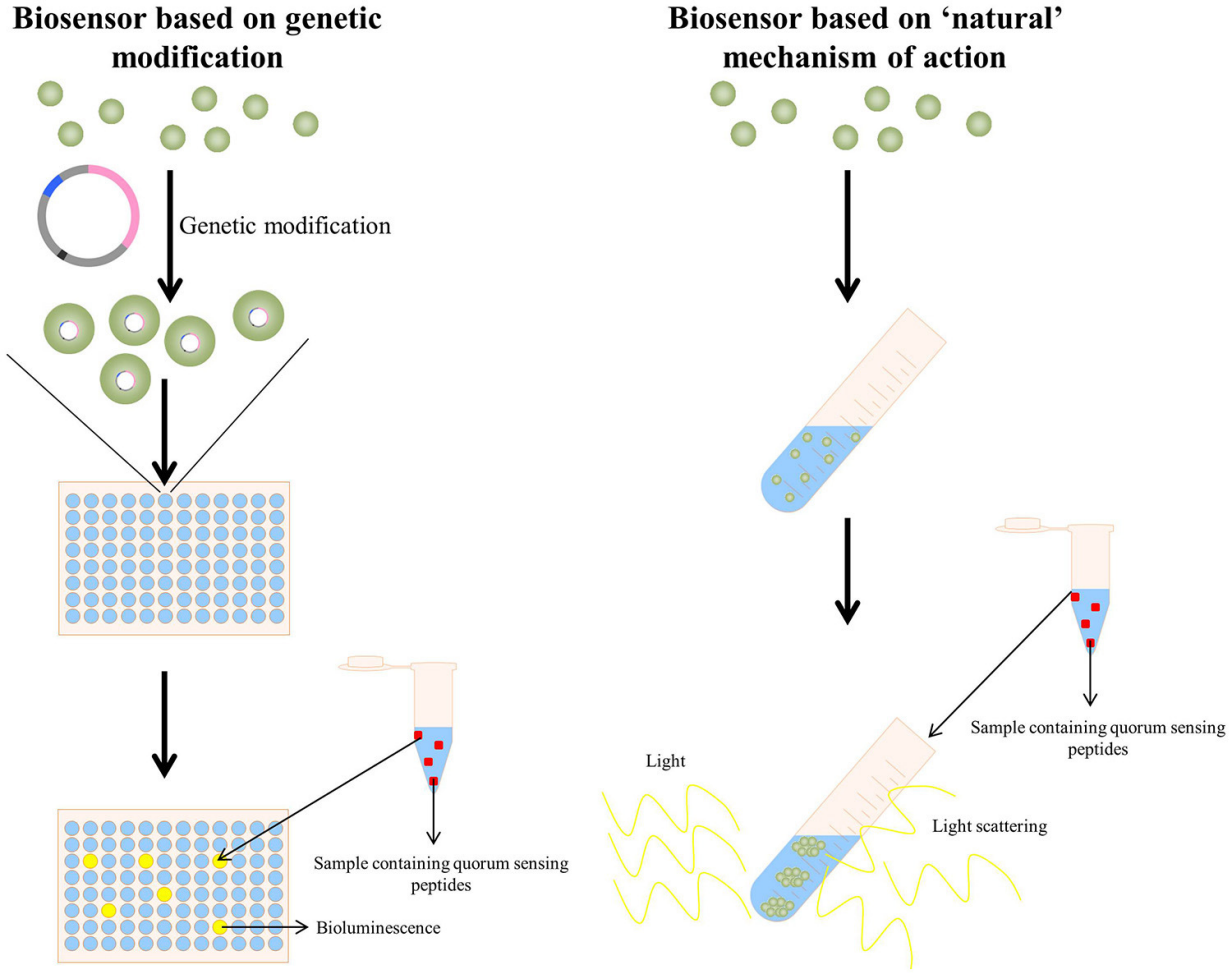
**(Left panel)** A quorum sensing reporter strain based on genetic modification: bacteria are genetically modified to make them competent to detect the specific quorum sensing peptide. Upon addition of a sample containing the quorum sensing peptide, the activated quorum sensing peptide receptor triggers in this example bioluminescence, allowing detection and quantification of the quorum sensing peptide (La Rosa et al., 2015). **(Right panel)** A quorum sensing reporter strain based on the naturally observed quorum sensing mechanism of a quorum sensing peptide (in this example the clumping induction assay as observed with various *E. faecalis* quorum sensing peptides Suzuki et al., 1984; Mori et al., 1988; Nakayama et al., 1995a). A sample containing the quorum sensing peptide is added to a bacterial strain capable of detecting the quorum sensing peptide and the formation of clumps is observed via light scattering.

Regarding quorum sensing bacterial reporters, two approaches are currently being used, i.e., (I) those based on the “natural” provoked effect by the specific quorum sensing peptide and (II) by applying genetically modified bacteria acting as detecting strains (Figure 3). The clumping assay (Suzuki et al., 1984; Mori et al., 1988; Nakayama et al., 1995a) can be considered as a system based on the “natural” provoked effects of some quorum sensing peptides: the bacteria applied as reporter strain are wild-type bacteria and have not been genetically altered to obtain receptiveness toward the specific quorum sensing peptide(s). When certain enterococcal quorum sensing peptides are added to receptive *Enterococci*, these quorum sensing peptides provoke clumping of the *Enterococci*. Consecutively, the turbidity of the bacterial suspension enhances and provides a quantifiable result via turbidimetry. The second type of bacterial reporters consists of bacteria that have been genetically modified to produce a signal in the presence of the quorum sensing molecule of interest; this type of reporter bacteria is more elaborately explained in the following sections.

#### Acyl homoserine lactones

Detection of quorum sensing molecules is often performed by using quorum sensing reporter bacteria strains, of which most are sensitive to either autoinducer-1 (AHL's) or autoinducer-2 (Steindler and Venturi, 2007; Rai et al., 2015). As mentioned previously, these bacterial reporters can be based on non-genetically modified or genetically-modified bacteria.

Genetic modifications consist of altering the bacterial genome, in order to obtain a detectable signal in the presence of the quorum sensing molecule of interest. This genetic modification consists of coupling a suitable promoter to a desired reporter gene, and hence the desired detection signal. The detection signal is produced by a reporter protein, e.g., bioluminescence, chemiluminescence, fluorescence, colorimetric response, etc. Production of the reporter protein depends on activation of the promoter, which initiates transcription of the reporter gene.

Inherent to genetic modifications of the bacteria, is the need of selecting those bacteria that are successfully genetically modified [e.g., those bacterial cells that have acquired the plasmid(s)]. Selecting these correctly, genetically modified bacteria can be performed by for example antibiotic resistance genes (O'Connor et al., 2016).

Table 1 provides an overview of plasmid-based AHL reporter bacteria.

**Table 1:** **Overview of some plasmid-based AHL reporter strains (Steindler and Venturi, 2007; Rai et al., 2015; O'Connor et al., 2016)**.

**Plasmid**	**Bacterial strain**	**Analyte**	**Promoter**	**Reporter**
pSB401	*E. coli*	3-oxo-C4- to 3-oxo-C14-HSL C4- to C12-HSL	*luxI*	*luxCDABE*
pSB1075	*E. coli*	3-oxo-C12- to 3-oxo-C16-HSL C12- to C16-HSL	*lasI*	*luxCDABE*
pECP61.5	*P. aeruginosa*	C4-HSL	*rhlA*	*lacZ*
pKDT17	*E. coli*	3-oxo-C10- to 3-oxo-C12-HSL C10- to C12-HSL	*lasB*	*lacZ*
pCF218, pMV26	*A.tumefaciens*	3-oxo-C6- to 3-oxo-C12-HSL C4- to C12-HSL	*traI*	*luxCDABE*
pCF218, pCF372	*A.tumefaciens*	3-oxo-C4- to 3-oxo-C12-HSL C5- to C10-HSL	*traI*	*lacZ*
pSB406	*E. coli*	3-oxo-C4- to 3-oxo-C14-HSL C4- to C12-HSL	*rhlI*	*luxCDABE*
	*C. violaceum*	3-oxo-C6- to 3-oxo-C8-HSL C4- to C8-HSL	*cviI*	Violacein
pAL105	*E. coli*	3-oxo-C12-HSL	*lasI*	*luxCDABE*
pAL101	*E. coli*	C4-HSL	*rhlI*	*luxCDABE*
pSB536	*E. coli*	C4-HSL	*ahyI*	*luxCDABE*
pSB403	*Broad host range*	3-oxo-C4- to 3-oxo-C14-HSL C4- to C12-HSL	*luxI*	*luxCDABE*
pHV2001	*E. coli*	3-oxo-C6- to 3-oxo-C8-HSL C6- to C8-HSL	*luxI*	*luxCDABE*
pZLR4	*A.tumefaciens*	All 3-oxo-HSLs C6- to C14-HSL 3-OH-C6- to 3-OH-C10-HSL	*traI*	*lacZ*
pJZ384, pJZ410, pJZ372	*A.tumefaciens*	3-oxo-C4- to 3-oxo-C18-HSL C4- to C18-HSL	*traI*	*lacZ*
pSF105, pSF107	*P. fluorescens*	3-OH-C6-HSL C6-HSL 3-OH-C8-HSL	*phzI*	*lacZ*
pUCP18	*P. aeruginosa*	3-oxo-C12-HSL	*rsaL*	*luxCDABE*
pMS402	*P. aeruginosa*	3-oxo-C12-HSL	*rsaL*	*luxCDABE*
pUCGMAT1-4	*E. coli*	3-oxo-C6-HSL	*ahlI*	*mcherry*
pREC-FF	*E. coli*	3-oxo-C6-HSL	*luxI*	*cfp*
M71LZ	*P. aeruginosa lasI ^−^*	3-oxo-C10 to 3-oxo-C12-HSL	*lasI*	*lacZ*
pAS-C8	*Broad host range*	C8- to C10-HSL	*cepI*	*gfp*
pKR-C12	*Broad host range*	3-oxo-C10- to 3-oxo-C12-HSL	*lasI*	*gfp*
pJBA-132	*Broad host range*	3-oxo-C6-HSL C6- to C10-HSL	*luxI*	*gfp*

#### Autoinducer-2

The *Vibrio harveyi* BB170 bioluminescence assay is an example of a reporter bacteria constructed by modifications of the bacterial genome to prevent production of and/or response to certain quorum sensing molecules. It is a frequently used reporter strain for quantification of autoinducer-2 molecules. Genetic modifications of the BB170 strain ensure it does not produce endogenous autoinducer-1 (AHL's) or autoinducer-2 and is insensitive for autoinducer-1. Hence, bioluminescence only occurs when autoinducer-2 is added, where the light intensity correlates with the amount of autoinducer-2 that is added (O'Connor et al., 2016).

#### Quorum sensing peptides

A variety of approaches are applied to proof the presence of, and in rare cases, eventually identify quorum sensing peptides (see Table 2). Currently, ~300 quorum sensing peptides and structural analogs have been published (Wynendaele et al., 2013). The majority of these peptides have been tested by the researchers on a system considered a reporter bacteria based biosensor system. While peptide structure elucidation was frequently performed by liquid chromatography-mass spectroscopy and/or Edman degradation. Analogs of the cognate quorum sensing peptides are also frequently evaluated using a specific biosensor.

**Table 2:** **Summary of quorum sensing peptide bio-sensor detection systems**.

**Quorum sensing molecule (amino acid sequence)**	**Species**	**LOD**	**Operational principle**
21-CSP (SGSLSTFFRLFNRSFTQALGK)	*S. mutans*	Not specified	β-galactosidase assay, Bacteriocin production, Transformation assay (Syvitski et al., 2007; Tian et al., 2009)
Gelatinase Biosynthesis-Activating Pheromone (QNSPNIFGQWM, lacton linkage between S3 and M11)	*E. faecalis*	Not specified	Bioluminescence (La Rosa et al., 2015)
EntF (AGTKPQGKPASNLVECVFSLFKKCN)	*E. faecium*	10 aM	Bacteriocin induction (Dunny et al., 1979)
cAM373 (AIFILAS)	*E. faecalis*	50 pM	Microtiter dilution method (Mori et al., 1986a)
cAD1 (LFSLVLAG)	*E. faecalis*	50 pM	Microtiter dilution method (Mori et al., 1984)
cCF10 (LVTLVFV)	*E. faecalis*	25 pM	Clumping induction assay (Mori et al., 1988)
cPD1 (FLVMFLSG)	*E. faecalis*	40 pM	Clumping induction assay (Suzuki et al., 1984)
cOB1 (VAVLVLGA)	*E. faecalis*	Not specified	Clumping induction assay (Nakayama et al., 1995a)
iPD1 (ALILTLVS)	*E. faecalis*	Not specified	Microtiter dilution method (Mori et al., 1987)
iAD1 (LFVVTLVG)	*E. faecalis*	Not specified	Microtiter dilution method (Mori et al., 1986b)
iAM373 (SIFTLVA)	*E. faecalis*	200 pM	Clumping induction assay (Nakayama et al., 1995b)
iCF10 (AITLIFI)	*E. faecalis*	10 nM	Inhibition of induced self-clumping (Nakayama et al., 1994)
CSP (DSRIRMGFDFSKLFGK)	*S. constellatus, S. anginosus, S. thermophilus*	0.2 nM	Biofilm formation assay (Petersen et al., 2004)
CSP (DRRDPRGIIGIGKKLFG)	*S. milleri*	200 ng/ml ≈ 105 nM	Transformation assay (Håvarstein et al., 1997)
CSP (SQKGVYASQRSFVPSWFRKIFRN)	*S. gordonii*	200 ng/ml ≈ 72 nM	Transformation assay (Håvarstein et al., 1997)
CSP (EMRISRIILDFLFLRKK)	*S. pneumoniae*	100 ng/ml ≈ 46 nM	Transformation assay (Pozzi et al., 1996)
CSP (EMRLSKFFRDFILQRKK)	*S. pneumoniae*	100 ng/ml ≈ 45 nM	Transformation assay (Pozzi et al., 1996)
EDF (NNWNN)	*E. coli*	<100 ng/ml ≈ 150 nM	Colony forming units assay (Kolodkin-Gal et al., 2007)

For example, two reporter strains in *S. mutans* GS5, capable of detecting the competence stimulating peptide (21-CSP) UA159sp4 have been described. The CSP-mediated quorum sensing sytem of *S. mutans* involves the genes *comCDE, comAB*, and *comX*. In brief, the CSP precursor, encoded by *comC*, is cleaved and exported to form CSP by an ABC-transporter encoded by *comAB*. Additionally, the *comDE* gene encodes a two-component signal transduction system that recognizes CSP and initiates a response. *ComX* encodes a sigma factor that regulates transcription of several genes needed for uptake of foreign DNA and the ComE protein is activated by autophosphorylation upon binding of CSP with the ComD histidine kinase receptor. Phosphorylated ComE activates target genes such as *comCDE, comAB*, and *comX*, thereby generating a positive feedback quorum sensing system (Syvitski et al., 2007). The reporter strain is composed of a *comC* mutant *S. mutans* GS5, resulting in a mutant strain which is unable to produce CSP itself, but still able to respond to exogenous CSP, named SMdC. Mutation of the *comC* gene is obtained by inserting a spectinomycin (*Specr*) resistance gene in the *comC* gene of *S. mutans* GS5. This mutant was used to develop two *lacZ* transcriptional reporter strains in which the *lacZ* gene is fused to the *comDE* promoter or the *nlmAB* promoter. To generate the first reporter strain, a vector pYH2 was formed by fusion of the *comDE* promoter with a promoterless *lacZ* gene in the *Streptococcus*—*Escherichia coli* shuttle vector pSL; subsequently, pYH2 was transformed into SMdC, resulting in the SMdC-pYH2 reporter strain. The second reporter strain is constructed by fusion of the *nlmAB* promoter with a promoter-lacking *lacZ* gene and transformation of this pOMZ47 plasmid into SMdC, resulting in the SMdC-PnlmAB reporter strain (Steindler and Venturi, 2007). Following addition of exogenous CSP (UA159sp), due to transcription of the *lacZ* gene, both SMdC-pYH2 and SMd-C-PnlmAB express β-galactosidase (Syvitski et al., 2007; Tian et al., 2009). Cleavage of O-nitrophenyl-β-D-galactoside by β-galactosidase renders O-nitrophenol, with the yellow color proportional to the enzyme activity and hence the quorum sensing peptide concentration (Syvitski et al., 2007).

The ability of CSP (0.2–100 nM) to induce competence in *S. intermedius* NCTC 11324 was tested: overnight cells of *S. intermedius* NCTC 11324 were diluted 1:200 and subsequently, quorum sensing peptide and a plasmid expressing erythromycin resistance genes were added. After incubation for 24 h, transformants were selected by growth on THB agar plates containing erythromycin. The amount of transformants/mL increased when increasing concentrations of CSP were added (Wynendaele et al., 2015a). Comparable transformation assays were performed by Håvarstein et al. (1997) with two other synthetic CSPs from *Streptococcus milleri* and from *Streptococcus gordonii* (Håvarstein et al., 1997) and by Pozzi et al. (1996).

The reporter strain for the Gram-negative *E. coli* quorum sensing peptide “extracellular death factor” (EDF) was described by Kolodkin-Gal et al. (2007). They studied the influence of the quorum sensing peptide on mazEF-mediated cell death in *E. coli*. The mazEF module encodes the stable toxin MazF, which exerts its toxic effects by cleavage of single-stranded mRNA at ACA sequences, and the labile antitoxin MazE, which counteracts the effect of MazF. The mazEF module is induced by stress. Stressful conditions that prevent transcription/translation of mazEF, such as inhibition of transcription and/or translation by antibiotics (e.g., rifampicin, chloramphenicol, spectinomycin) or DNA damage, reduces the level of the labile MazE, hereby reducing the counteracting effect on MazF. However, mazEF-mediated cell death only occurs in dense cultures since it requires the quorum sensing peptide EDF. Addition of rifampicin provokes mazEF-mediated cell death in dense cultures, but not in diluted cultures. The mazEF-mediated cell death in these diluted cultures could be restored by addition of supernatant of a dense culture. The bioassay for detection and quantification of EDF activity is based on this principle. Different dilutions of the supernatant of a dense culture of the *E. coli* strain MC4100relA+, containing EDF, were added to a diluted culture of MC4100relA+, after which stressful conditions (e.g., addition of chloramphenicol) were induced. The percentage of surviving colony forming units (CFUs) was determined after overnight incubation (Kolodkin-Gal et al., 2007).

Quorum sensing peptides can also be detected quantitatively (limit of detection 10 aM) by demonstrating a product of which formation is regulated by a quorum sensing peptide. Nilsen et al. (1998) studied the production of the bacteriocins enterocin A and enterocin B in *Enterococcus faecium* CTC492, regulated by the quorum sensing peptide EntF. Without EntF, a non-bacteriocin-producing culture (Bac–) of *Enterococcus faecium* CTC492 was obtained. Upon addition of induction factor EntF, production of enterocin A and enterocin B is induced and after 20–24 h incubation, bacteriocin activity is assayed by addition of sterilized supernatant to a culture of an indicator organism, e.g., *Lactobacillus sakei* NCDO 2714 (sensitive to both enterocin A and enterocin B), *Pediococcus pentosaceus* FBB 63 (sensitive to enterocin A) or *Lactobacillus sakei* FVM 148 (sensitive to enterocin B). Growth inhibition of these strains indicates presence of bacteriocins in the supernatant and thus, presence of induction factor EntF in the sample added to the non-bacteriocin-producing culture (Nilsen et al., 1998).

A different approach uses the phenomenon of plasmid transfer to demonstrate the presence of quorum sensing peptides. Genes located on plasmids and encoding for hemolysins, bacteriocins, and antibiotic resistance genes are transferable. Several *Enterococcus faecalis* strains are able to transfer plasmids, facilitated by sex pheromones (i.e., quorum sensing peptides) (Dunny et al., 1979). Certain quorum sensing peptides, excreted by recipient strains, induce donor strains, containing conjugative plasmids, to produce a proteinaceous substance on their surface. Thereby, mating aggregates are formed, facilitating plasmid transfer. These quorum sensing peptides are also called clumping-inducing agents (CIA) since they cause self-aggregation of donor cells when culture filtrate of recipients is added (Dunny et al., 1979). Mori et al. (1984, 1986a, 1988), Suzuki et al. (1984) and Nakayama et al. (1995a), isolated and determined the sequence of several of these CIAs. Once the recipient strain acquires the conjugative plasmid, the excretion of the specific CIA, corresponding with the acquired plasmid, stops and the strain becomes sensitive to exogenous quorum sensing peptides. However, production of other CIAs, specific for donor strains containing other conjugative plasmids, still continues (Ike et al., 1983). Quantification of CIA can be performed by means of a microtiter system. Serial dilutions of recipient strain filtrates (or sample) are added to responder cells (donor cells with the appropriate conjugative plasmid) and incubated for 90–120 min, after which clumping is observed in dilutions (samples) containing sufficient amount of CIA (quorum sensing peptide) (Dunny et al., 1979). The same principle can be applied to other quorum sensing peptides facilitating plasmid transfer in *E. faecalis* e.g., cAD1 (Mori et al., 1984), cCF10 (Mori et al., 1988), cPD1 (Suzuki et al., 1984), and cOB1 (Nakayama et al., 1995a). Quorum sensing peptides cOB1 and cAD1 are an exception to the assumption of one pheromone (cX) specifically activating plasmid transfer of its corresponding plasmid (pX) since both pOB1 and pY11 respond to cOB1 and both pBEM10 and pAD1 respond to cAD1 (Murray et al., 1988; Nakayama et al., 1995a).

The production stop of quorum sensing peptide, once the recipient strain acquires the corresponding plasmid, is due to the production of a quorum sensing inhibitor encoded on the plasmid, which inhibits the activity of its corresponding quorum sensing peptide (Mori et al., 1987). Inhibitory activity of iPD1 (Mori et al., 1986b, 1987) and iAM373 (Nakayama et al., 1995b) can be quantitatively measured by modifying the microtiter system described above. Using iAD1 as example, serially diluted sample solutions containing iAD1 are added to a mixture of responder cells OG1S(pAD1::Tn917) and cAD1. After incubation for 2–3 h, inhibition of cAD1-induced clumping of responder cells is observed (Mori et al., 1986b).

### Chromatographic techniques

#### Liquid chromatography

A variety of liquid-chromatographic methods have been reported, especially for the *N*-acyl homoserine lactones (see Table 3) (Morin et al., 2003; Li et al., 2006; Fekete et al., 2007; Kumari et al., 2008; Ortori et al., 2011; Wang et al., 2011; Cataldi et al., 2013), including methods allowing the simultaneous analysis of various *N*-acyl homoserine lactones via a non-targeted LC-MS/MS method (Patel et al., 2016). Generally, these methods are capable of generating limits of detection (LOD) in the broad nM-range (median LOD of 2.3 nM for 19 *N*-acyl homoserine lactone standards; range: 0.6 nM–2838.9 nM), with some compounds owing a limit of detection around 0.6 nM (for C10-HSL) (Patel et al., 2016).

**Table 3:** **Summary of reported concentrations of quorum sensing molecules in different matrices**.

**Quorum sensing molecule**	**Matrix**	**Detection method**	**Concentration**
**AUTOINDUCER-2**			
AI-2	S. oralis 34 *in vitro* biofilm	*V. harveyi* BB170 bioluminescence assay	69–123 nM over 48 h (Rickard et al., 2008)
	*A. naeslundii* T14V *in vitro* biofilm	*V. harveyi* BB170 bioluminescence assay	153–382 nM over 48 h (Rickard et al., 2008)
	*S. oralis* 34 and *A. naeslundii* T14V *in vitro* dual-species biofilm	*V. harveyi* BB170 bioluminescence assay	124–197 nM over 48 h (Rickard et al., 2008)
	*S. oralis* 34 *luxS* mutant and *A. naeslundii* T14V *in vitro* dual-species biofilm	*V. harveyi* BB170 bioluminescence assay	122–140 nM over 48 h (Rickard et al., 2008)
	Saliva (*n* = 8)	HPLC-MS	244–965 nM (mean = 526 nM) (Campagna et al., 2009)
	Saliva IBD patients (*n* = 3)	*V. harveyi* BB170 bioluminescence assay	1.67–2.29 μM (mean = 2.05 μM) (Raut et al., 2013)
	Stool IBD patients (*n* = 2)	*V. harveyi* BB170 bioluminescence assay	1.57–3.59 μM (mean = 2.76 μM) (Raut et al., 2013)
	Ileal washing IBD patients (*n* = 1)	*V. harveyi* BB170 bioluminescence assay	2.29 μM (Raut et al., 2013)
	Saliva healthy volunteers (*n* = 3)	*V. harveyi* BB170 bioluminescence assay	0.998–2.07 μM (mean = 1.39 μM) (Raut et al., 2013)
**AHL**			
3-oxo-C12-HSL	Sputum samples (*n* = 47) (20 CF patients + 2 healthy volunteers)	LC-MS	20.24–6833.20 nM (Struss et al., 2013)
	P. aeruginosa *in vitro* biofilms (*n* = 4)	LC-MS	Mean = 0.95 ± 0.68 μM (Struss et al., 2013)
	Sputum CF patients (*n* = 38/59)	LC-MS/MS	LLOQ (not specified)–410 nM (Barr et al., 2015)
	Sputum CF patients (*n* = 10/12)	pKDT17 with *lasB:lacZ* fusion in *E. coli*	0.92–21.20 nM (mean = 5.04 nM) (Marchesan et al., 2015)
HHQ	Sputum CF patients (*n* = 35/59)	LC-MS/MS	LLOQ (not specified)–1066 nM (Barr et al., 2015)
	Plasma CF patients (*n* = 40/56)	LC-MS/MS	0.01 nM (i.e., LLOQ)–2.744 nM (Barr et al., 2015)
	Urine CF patients (*n* = 31/59)	LC-MS/MS	0.02 nM (i.e., LLOQ)–4.028 nM (Barr et al., 2015)
NHQ	Sputum CF patients (*n* = 43/59)	LC-MS/MS	LLOQ (not specified)–1563 nM (Barr et al., 2015)
	Plasma CF patients (*n* = 25/56)	LC-MS/MS	0.01 nM (i.e., LLOQ)–0.222 nM (Barr et al., 2015)
	Urine CF patients (*n* = 19/59)	LC-MS/MS	0.01 nM (i.e., LLOQ)–0.347 nM (Barr et al., 2015)
HQNO	Sputum CF patients (*n* = 47/59)	LC-MS/MS	LLOQ (not specified)–780 nM (Barr et al., 2015)
	Plasma CF patients (*n* = 21/56)	LC-MS/MS	0.03 nM (i.e., LLOQ)–0.904 nM (Barr et al., 2015)
	Urine CF patients (*n* = 48/59)	LC-MS/MS	0.03 nM (i.e., LLOQ)–12.511 nM (Barr et al., 2015)
NQNO	Sputum CF patients (*n* = 26/59)	LC-MS/MS	LLOQ (not specified)–1075 nM (Barr et al., 2015)
	Plasma CF patients (*n* = 15/56)	LC-MS/MS	0.04 nM (i.e., LLOQ)–0.705 nM (Barr et al., 2015)
	Urine CF patients (*n* = 16/59)	LC-MS/MS	0.05 nM (i.e., LLOQ)–2.067 nM (Barr et al., 2015)
C7-PQS	Sputum CF patients (*n* = 21/59)	LC-MS/MS	LLOQ (not specified)–873 nM (Barr et al., 2015)
	Plasma CF patients (*n* = 8/56)	LC-MS/MS	0.1 nM (i.e., LLOQ)–0.571 nM (Barr et al., 2015)
C9-PQS	Sputum CF patients (*n* = 16/59)	LC-MS/MS	LLOQ (not specified)–4302 nM (Barr et al., 2015)
C4-HSL	Sputum CF patients (*n* = 18/59)	LC-MS/MS	LLOQ (not specified)–145 nM (Barr et al., 2015)
	Sputum CF patients (*n* = 6/12)	pECP61.5 with *rhlA:lacZ* fusion in *P. aeruginosa*	0.84–5.00 nM (mean = 1.04 nM) (Erickson et al., 2002)
C10-HSL	*Burkholderia cepacia* JA-8 cell culture broth (*n* = 4)	LC-MS/MS	260 nM (9% RSD) (Frommberger et al., 2004)
C8-HSL	*Burkholderia cepacia* JA-8 cell culture broth (*n* = 4)	LC-MS/MS	180 nM (12% RSD) (Frommberger et al., 2004)

Besides the closely related cyclodipeptides demonstrated qualitatively, the presence of quorum sensing peptides has not yet been demonstrated *in vivo* (Marchesan et al., 2015), except for one report (Darkoh et al., 2015) where the presence of a thiolacton peptide, without structural elucidation, is suggested in feces of patients suffering from *C. difficile* infection. Nevertheless, the presence of these peptides in cell culture broths has already been proven via reporter strains (see Table 2). Additionally, various authors have reported quorum sensing peptide levels in bacterial cell culture broths via chromatographic techniques, hence applying an orthogonal detection method compared to the biological reporter bacteria.

Additional to reporter bacteria based biosensors, several quorum sensing peptides have been qualitatively or quantitatively determined in bacterial cell culture broths (see Table 4). For example, the currently reported *E. faecalis* quorum sensing peptides have been quantified in cell culture broths. Based on the reports, the concentration of these quorum sensing peptides is in the broad picomolar range, though caution should be paid to the often elaborate sample preparation methods that have been applied, mostly intended for a qualitative approach.

**Table 4:** **Concentration of selected peptides in bacterial cell culture broths**.

**Species**	**Quorum sensing peptide (sequence)**	**Strain**	**Concentration**	**Detection method**
*Enterococcus faecalis*	cAM373 (AIFILAS)	*E. faecalis* JH2-2 (pAM351)	4.4 μg/16 L = 374 pM	HPLC-UV/VIS (Mori et al., 1986a)
	iPD1 (ALILTLVS)	*E. faecalis* JH2-2 (pAM351)	6.3 μg/20 L = 378 pM	HPLC-UV/VIS (Mori et al., 1987)
	iCF10 (AITLIFI)	*E. faecalis* OG1RF(pMSP5011)	15,360 pg/mL = 19,440 pM	Estimated via bio-assay via comparing with synthetic peptide (La Rosa et al., 2015)
	cOB1 (VAVLVLGA)	*E. faecalis* FA2-2	1.5 μg/15 L = 135 pM	HPLC-UV/VIS (Nakayama et al., 1995a)
	cAD1 (LFSLVLAG)	*E. faecalis* JH2-2	200 μg/300 L = 814 pM	HPLC-UV/VIS (Mori et al., 1984)
	iAD1 (LFVVTLVG)	*E. faecalis* FA2-2 (pAM727)	20 μg/20 L = 787 pM	HPLC-UV/VIS (Mori et al., 1986b)
	cCF10 (LVTLVFV)	*E. faecalis* FA2-2 (pAM351)	4.1 μg/60 L = 86 pM	HPLC-UV/VIS (concentration) FAB-MS (amino acid sequence confirmation) (Mori et al., 1988)
	iAM373 (SIFTLVA)	*E. faecalis*JSS2 (pAM377)	3 μg/8 L = 500 pM	HPLC-UV/VIS (Nakayama et al., 1995b)

Besides the presence of *E. faecalis* quorum sensing peptides in cell culture broths, other authors have developed chromatographic methods to demonstrate quorum sensing peptide presence of other species in their respective cell cultures (Junio et al., 2013; Todd et al., 2016). For example, the presence of *Staphylococcus* autoinducing peptide I was demonstrated using a ultra-high performance liquid chromatographic system coupled to a mass spectrophotometer in methicillin-resistant *Staphylococcus aureus* cell cultures with a limit of detection of 3.5 nM and a LOQ of 0.10 μM (Todd et al., 2016). Junio et al. (2013) demonstrated quantitatively (LOD 0.25 μM; LOQ 2.6 μM) the time- and strain dependent synthesis of AIP-1 in cell culture medium in the μM-magnitude, with concentrations as high as 13 ± 2 μM at 16 h incubation of the *S. aureus* LAC strain (Junio et al., 2013).

Additionally, the extracellular death factor quorum sensing peptide from *E. coli* has also been demonstrated in cell culture broths via a qualitative chromatographic method (Kolodkin-Gal et al., 2007).

Quorum sensing is not limited to bacteria, but eukaryotic yeasts have also shown to possess quorum sensing mechanisms. The *C. albicans* quorum sensing molecules farnesol and tyrosol are via a LC-MS/MS method quantifiable with limits of detection of respectively 15.2 and 3.0 nM (Gregus et al., 2010).

#### Gas chromatography

Via gas chromatography coupled to a mass spectrometer (GC-MS), an enantiomer-selective method was achieved for the analysis of C8-HSL (LOD: 0.1 mg/L ≈ 0.4 μM for L-C8-HSL), C10-HSL (LOD: 0.1 mg/L ≈ 0.4 μM for L-C10-HSL), and C12-HSL (LOD: 0.3 mg/L ≈ 1.1 μM for L-C12-HSL). By applying this method to *Burkholderia cepacia* LA3 cell culture supernatant, the authors demonstrated the *in vitro* production of D-C10-HSL (Malik et al., 2009). *N*-3-oxoacyl homoserine lactones were determined by derivatizing them into their pentafluorobenzyloxime derivatives with subsequent gas chromatography-mass spectrometry analysis. ODHL (biofilm: 3 ± 2 μM; effluent: 1 ± 0.1 nM), OdHL (biofilm: 632 ± 381 μM; effluent: 14 ± 3 nM), OtDHL (biofilm: 40 ± 15 μM; effluent: 1.5 ± 0.7 nM), and OOHL (effluent: 0.1 ± 0.1 nM) were by applying this method found in biofilms and/or effluent (Charlton et al., 2000). Cataldi et al. (2007) also reported a GC-MS method capable of detecting AHL's in the μM-range with an average LOD of 4.4 μM for the 6 investigated AHLs (Cataldi et al., 2007). The autoinducer-2, following silylation, has also proven to be analyzable by gas chromatography-mass spectrometry (LOD 5.3 nM and limit of quantification (LOQ) 16 nM) (Thiel et al., 2009).

#### Thin-layer chromatography

Besides liquid and gas chromatography, (high performance-) thin-layer chromatography has also been used in the analysis of certain quorum sensing molecules (Shaw et al., 1997; Bala et al., 2013). Shaw et al. (1997) achieved LODs ranging from 0.5 fmol to 300 pmol depending on the specific AHL (Shaw et al., 1997), whereas Bala et al. (2013) reported a lower limit of detection of 0.006 nmol/spot and a lower limit of quantification of 0.01 nmol/spot for PQS (Bala et al., 2013). A hybrid technique, combining bacterial reporters with thin-layer chromatography has been described (Andersen et al., 2001; Charlesworth et al., 2015) (LODs ranging from 0.012 to 1,710 ng depending on the specific AHL) (Charlesworth et al., 2015).

#### Capillary electrophoresis

Besides liquid and gas chromatography, some authors reported capillary electrophoresis as a suitable technique to quantify quorum sensing molecules. With partial filling micellar electrokinetic chromatography–electrospray ionization-ion trap mass spectrometry, two acyl homoserine lactone derivatives (i.e., *N*-octanoyl-L-homoserine lactone and *N*-decanoyl-L-homoserine lactone) molecules originating from *Burkholderia cepacia* could be qualitatively detected in bacterial cell culture broths down to a concentration of approximately 1 μM (Frommberger et al., 2003). The same authors were capable of determining quantitatively (LOQ 0.05 μg/ml) C8-HSL (0.26 μg/ml ≈ 1.1 μM), C10-HSL (1.93 μg/ml ≈ 7.6 μM), and C12-HSL (0.16 μg/ml ≈ 0.6 μM) in *Burkholderia cepacia* cell culture broths by means of capillary zone electrophoresis-mass spectrometry following hydrolysis of the homoserine lactones into their corresponding organic acids (Frommberger et al., 2005).

Several *P. aeruginosa*-quinolones are also detectable using capillary electrophoresis with LODs of 65 nM (PQS), 94 nM (HHQ), 61 nM (“Quinolone 1”) and 79 nM (“Quinolone 2”) (Zhou et al., 2012). Noteworthy is the eukaryotic quorum sensing molecule from *C. albicans* farnesol, which is also detectable via capillary electrophoresis. By applying this capillary electrophoresis method, the authors demonstrated the concentration of farnesol to be approximately 2 nM in a cell culture supernatant (Kubesová et al., 2010).

### Miscellaneous analytical techniques

Cyclic voltammetry and amperometric detection by a bare boron-doped diamond electrode showed, however with selectivity limitations, to be a possible approach to detect 2-heptyl-3-hydroxy-4-quinolon from *P. aeruginosa* at approximately 30 μM in *P. aeruginosa pqsL*^−^ mutant strains cell culture medium (Zhou et al., 2011).

Other authors also developed electrochemical biosensors for the detection of AHL's, achieving detection limits down to 2 pM via cyclic voltammetry with a gold microelectrode (Baldrich et al., 2011).

Zhang and Ye (2014) reported a Förster resonance energy transfer-based biosensor for the detection of *N*-(3-oxo-hexanoyl)-L-homoserine lactone. However, the reported method had a limit of detection as high as 100 μM (Zhang and Ye, 2014).

Enzyme-linked immunosorbent assay (ELISA) also proved a suitable approach for AHL-detection. Chen et al. (2010a) developed rat monoclonal antibodies achieving limits of detection of 31 nM concerning 3-oxo-C10-HSL and 476 nM regarding C8-HSL (Chen et al., 2010a). Additionally, Kaufmann et al. (2006) developed an antibody against 3-oxo-C12-HSL (Kaufmann et al., 2006) without aiming to develop an ELISA. However, this antibody could still be suitable for ELISA and has according to Chen et al. (2010a) a limit of detection of 15 nM (Chen et al., 2010a). Another ELISA method has been developed to monitor AHL production in *B. cepacia* LA3 cell cultures (Chen et al., 2010b).

Finally, surface-enhanced Raman spectroscopy also proved a feasible approach for AHL detection with a LOD estimated around 3 nM (Pearman et al., 2007).

## *In vivo* concentration of quorum sensing molecules

### Autoinducer-2

*Streptococcus oralis, Streptococcus oralis luxS* mutant, and *Actinomyces naeslundii* T14V each produce autoinducer-2 when they are grown in commercial media (Rickard et al., 2006). Rickard et al. (2006, 2008) investigated if each of these species also produce autoinducer-2 when they are part of single- or dual-species biofilms in saliva in an *ex-vivo in-vitro* design. Biofilms were grown within Sorbarods fed with 25% human saliva, after which the autoinducer-2 concentration in the effluent was determined by the *Vibrio harveyi* BB170 bioluminescence assay. Autoinducer-2 concentrations in a single-species biofilm of *S. oralis* 34 or *A. naeslundii* T14V varied between 69–123 and 153–382 nM over 48 h respectively. In the effluent from the *S. oralis* 34 *luxS* mutant biofilm, no autoinducer-2 could be detected. Autoinducer-2 concentrations in the effluent of dual-species biofilms containing *S. oralis* 34 and *A. naeslundii* T14V or *S. oralis* 34 *luxS* mutant and *A. naeslundii* T14V varied respectively between 124–197 and 122–140 nM over 48 h (Rickard et al., 2006, 2008).

Campagna et al. (2009) developed a HPLC-MS method (LOD in the low nM-range) for the detection of autoinducer-2 in human saliva. They asked 8 volunteers to donate approximately 1 ml of saliva and determined the concentration of autoinducer-2 in these samples. The average concentration was found to be 526 nM with individual values ranging from 244 to 965 nM (Campagna et al., 2009).

Autoinducer-2 concentrations in saliva, stool and intestinal samples from patients with irritable bowel disease (IBD), as well as in saliva samples from healthy volunteers, were determined using the *Vibrio harveyi* BB170 bioluminescence assay. Because of genetic modifications, the BB170 strain only emits light when autoinducer-2 is added. Dose-response curves were obtained using 50 nM–0.5 mM dilutions of synthetic autoinducer-2 in saliva or stool matrix. The autoinducer-2 concentration was quantified in stool (*n* = 2, 2.76 μM), ileal washing samples (*n* = 1; 2.29 μM) and saliva from patients with IBD (*n* = 3, 2.05 μM) and saliva from healthy volunteers (*n* = 3; 1.39 μM) (Raut et al., 2013).

An overview of the observed *in vivo* concentrations is given in Table 3.

### Acyl homoserine lactones

Chronic lung disease in cystic fibrosis patients is frequently associated with *Pseudomonas aeruginosa* biofilm formation in the lungs (Singh et al., 2000). Quorum sensing in *P. aeruginosa* is mediated via *N*-3-oxododecanoyl homoserine lactone and *N*-butyryl homoserine lactone (Pesci et al., 1997). *N*-3-oxododecanoyl homoserine lactone is indispensable for the establishment of a persistent *P. aeruginosa* infection; therefore a LC-MS method for the quantitation of this quorum sensing molecule in sputum of cystic fibrosis patients has been developed (LLOQ of 10 nM for 3-oxo-C12-HSL) (Struss et al., 2013). In total, 47 samples originating from 20 cystic fibrosis and 2 healthy donors were collected. Concerning the cystic fibrosis patients, 9 patients donated more than 1 sample, resulting in 34 samples from these patients obtained in total at different time points. Cystic fibrosis patients were allocated into different groups depending on their disease state. Besides patient samples, they also investigated the *N*-3-oxododecanoyl homoserine lactone concentration in *P. aeruginosa in vitro* cultured biofilms (*n* = 4) and found a mean concentration of 0.95 ± 0.68 μM. Regarding the patient samples, 45 out of 47 samples contained *N*-3-oxododecanoyl homoserine lactone in quantifiable concentrations with concentrations ranging between 20 to > 1,000 nM; the highest measured concentration was approximately 6,900 nM (Struss et al., 2013). These results are potentially clinically significant as *N*-3-oxododecanoyl homoserine lactone in a concentration range of 0.1–100 μM has shown to alter the human immune response (Telford et al., 1998b).

Barr et al. (2015) determined the concentration of several quorum sensing molecules in sputum, plasma and urine of 60 cystic fibrosis patients with chronic *Pseudomonas aeruginosa* infection at the beginning of a pulmonary exacerbation (Barr et al., 2015). All samples were analyzed by liquid chromatography—tandem mass spectrometry (LC-MS/MS). Eight different quorum sensing molecules were observed in sputum: 2-heptyl-4-hydroxyquinoline (HHQ), 2-nonyl-4-hydroxyquinoline (NHQ), 2-heptyl-4-hydroxyquinoline-*N*-oxide (HQNO), 2-nonyl-4-hydroxyquinoline-*N*-oxide (NQNO), 2-heptyl-3-hydroxy-4(1*H*)-quinolone (C7-PQS), 2-nonyl-3-hydroxy-4(1*H*)-quinolone (C9-PQS), *N*-3-oxododecanoyl-L-homoserine lactone (3-oxo-C12-HSL) and *N*-butanoyl-L-homoserine lactone (C4-HSL). Five of them were also detected in plasma (HHQ, NHQ, HQNO, NQNO, and C7-PQS) and four in urine samples (HHQ, NHQ, HQNO, NQNO). Concentrations in sputum, plasma, and urine varied respectively between lower limit of quantification (LLOQ) (not specified)–4,302 nM, LLOQ (i.e., 0.01 nM)–2.744 nM and LLOQ (i.e., 0.01 nM)–12.511 nM and do correlate with the disease state of cystic fibrosis (Barr et al., 2015).

The concentration of 3-oxo-C12-HSL and C4-HSL in sputum samples of 12 patients with cystic fibrosis has been assessed. In order to determine the 3-oxo-C12-HSL concentration, a pKDT17 plasmid in *E. coli*, which contains a copy of the *lasR* gene and a *lasB-lacZ* fusion, was used. Concentrations of C4-HSL were determined by means of a pECP61.5 plasmid in *P. aeruginosa*, which contains a copy of the *rhlR* gene and a *rhlA-lacZ* fusion. The measured β-galactosidase activity in both reporter bacteria correlates with the levels of respectively 3-oxo-C12-HSL and C4-HSL. The average concentrations of 3-oxo-C12-HSL and C4-HSL were found to be respectively 5.04 and 1.04 nM with individual values ranging from < LOD (not specified) to 21.2 nM and < LOD (not specified) to 5 nM. However, determination of the extraction efficiency by adding synthetic autoinducers to sputum samples that do not contain autoinducer showed only 80% recovery of 3-oxo-C12-HSL and 60–65% recovery of C4-HSL, indicating loss of autoinducer during extraction in sample preparation (Erickson et al., 2002).

A summary of the reported concentrations of quorum sensing molecules in different matrices, together with the method used for detection and the corresponding reference is given in Table 3. To our knowledge, quorum sensing peptides have not yet been quantitated nor identified in biological samples from human origin.

## Discussion

### Detection methods

A variety of analytical techniques is currently available for many quorum sensing molecules, mainly chromatographic methods and reporter bacteria based biosensors. However, caution must be paid when considering these different methods regarding their analytical characteristics like selectivity. The reporter bacteria are based on the interaction of the molecule of interest with a receptor. Hence, structural analogs can possibly also interact with the receptor and consecutively might impede a straight-forward quantitative or qualitative selective analysis of individual quorum sensing molecules, a rather general concern that is also valid for other analytical techniques. For example, applying ELISA as a quantitative analytical method, cannot exclude cross-reactivity toward other structurally analog quorum sensing molecules that are currently not yet discovered. Since these molecules currently might not yet have been identified, it is very difficult to test for cross-reactivity toward these molecules. This can be illustrated by a variety of quorum sensing peptides listed in the Quorumpeps database: various synthetic structural analogs of certain quorum sensing peptides have been tested toward the bacteria receptive for the cognate quorum sensing peptide. The reactivity of the receptors is not solely limited toward synthetic analogs: the receptor(s) have been proven to be activated by various naturally occurring structural analogs (e.g., the *N*-acyl homoserine lactones). This potential pitfall can be omitted by coupling the biosensor with an initial chromatographic separation by e.g., thin layer chromatography. On the other hand, mass spectroscopy, especially in its targeted modi, allows additional selectivity via quorum sensing molecule fragmentation. Another important method consideration for qualitative and/or quantitative analysis of samples is the achievable limit of detection and its related, always higher, limit of quantification. This analytical characteristic is depending on the analytical method as such, including the sample preparation steps and is also compound-dependent. The latter being illustrated by the large range of limits of detection reported for various methods. The bacteria reporter based biosensors have in some specific cases proven to achieve limits of detection and limits of quantification in the sub-pM range. Chromatography coupled to mass spectroscopy can also achieve limits of detection and quantification in the sub-nM or even below. Hence, the preferred method of detection and/or quantification mainly depends on the desired selectivity and sensitivity for a specific quorum sensing molecule or group of molecules.

### *In vivo* quorum sensing molecule concentrations

The *in vivo* presence and concentration of quorum sensing peptides have been neglected, while the other classes of quorum sensing molecules (e.g., the *N*-acyl homoserine lactones) have attracted considerable attention. The concentration of these molecules has been demonstrated in both healthy and diseased subjects. Concentrations of AI-2 were found to be in the high nM–low μM range and AHL concentrations were found in a comparable concentration range.

The lack of information related to the large group of quorum sensing peptides in *in vivo* samples cannot be longer justified. Peptides play an important role in all aspects of the immune, endocrine, and neuronal systems, and hence, the biological roles of quorum sensing peptides as produced by our microbiome are expected to be elucidated in the near future. Moreover, taking into consideration the increasing evidence that our microbiome is involved in a variety of biological host processes, both in health as in disease, these quorum sensing peptides could act as biomarkers, similar to recent suggestions of using microbiota as biomarkers (Eloe-Fadrosh and Rasko, 2013). This can be illustrated by the reports of quorum sensing molecules in cystic fibrosis patients (Singh et al., 2000).

## Conclusion

Quorum sensing molecules are a heterogeneous group of molecules currently only started to be explored in *in vivo* situations. For example, to our knowledge, the bacterial quorum sensing peptides have sparsely qualitatively or quantitatively detected *in vivo* within humans. Conversely, the *N*-acyl homoserine lactones do have attracted considerable scientific interest. They have been quantified *in vivo* at concentrations ranging in the broad nM-μM range. Quorum sensing peptides have, together with the other classes of quorum sensing molecules, been detected and quantified in bacterial cell culture broths. The concentration of these quorum sensing molecules is highly variable and *in vitro* observed concentrations can reach almost mM concentrations.

A variety of analytical techniques has been described in literature to investigate the major quorum sensing molecule classes. Generally, reporter bacteria based biosensors and chromatography, especially LC(-MS/MS) are most frequently applied for qualitative and quantitative purposes. Selectivity and sensitivity are key analytical characteristics when considering quorum sensing molecule identification and quantification. Concerning selectivity, reporter bacteria based biosensors will potentially be confronted with cross-reactivity, and hence potential false-positives, due to currently unknown quorum sensing molecules that might cross-react with the specific quorum sensing molecule receptor. However, these reporter bacteria biosensors are, in some specific cases, able to achieve very low limits of detection (e.g., pM range), sometimes outperforming the LC(-MS/MS) methods. Opposite, LC-MS/MS methods potentially show a higher degree of selectivity due to both the chromatographic separation and the molecule fragmentation patterns via mass spectroscopy. Hence, the preferred method of analysis does depend on the quorum sensing molecule(s) of interest and the objectives put forward.

Evidence of the role of the human microbiome, both in health and disease, is continuously increasing. Quorum sensing molecules are bacterial languages, and apart from their fundamental biological importance in pathophysiological conditions, they can serve as potential prognostic or diagnostic biomarkers. However, the vast potential field of quorum sensing molecule-biomarkers, especially quorum sensing peptides, have not yet received the attention they deserve.

## Author contributions

FV and BD conceived the idea for this manuscript, while FV and SD wrote the manuscript, FV, SD, ND, YJ, EW, CV, and BD made substantial contributions to data interpretation and drafted/revised the manuscript. FV, SD, ND, YJ, EW, CV, and BD approved this manuscript and agree to be held accountable for all aspects of this manuscript.

## Funding

FV is funded by the “Institute for the Promotion of Innovation through Science and Technology in Flanders (IWT-Vlaanderen)” (Grant number 131356 to FV). ND is funded by “Fonds wetenschappelijk onderzoek-Vlaanderen (Fund Scientific Research-Flanders)” (Grant number 1S21017N to ND). IWT-Vlaanderen or Fonds wetenschappelijk onderzoek-Vlaanderen were not involved in any part of the study, and had no role in the conception, contributions to the consent nor in manuscript writing.

### Conflict of interest statement

The authors declare that the research was conducted in the absence of any commercial or financial relationships that could be construed as a potential conflict of interest. The reviewer SOF and handling Editor declared their shared affiliation, and the handling Editor states that the process nevertheless met the standards of a fair and objective review
